# Phenotypic and genomic study of digital dermatitis in UK Holstein heifers

**DOI:** 10.1186/s12864-026-13016-y

**Published:** 2026-06-10

**Authors:** Eirini Tarsani, Bingjie Li, Alkiviadis Anagnostopoulos, Ana Sato, Kaixin Bao, Amy Gillespie, Nektarios Siachos, Lara Robinson, Mike Coffey, Androniki Psifidi, Georgios Oikonomou, Georgios Banos

**Affiliations:** 1 Scotland’s Rural College (SRUC), The King’s Buildings, West Mains Road, Edinburgh, EH9 3JG UK; 2 Department of Livestock and One Health, Institute of Infection, Veterinary and Ecological Sciences, University of Liverpool, Leahurst Campus, Neston, CH64 7TE UK; 3 Scotland’s Rural College (SRUC), School of Veterinary Medicine, Ferguson Building, Craibstone Estate, Aberdeen, AB21 9YA UK; 4 Royal Veterinary College, Hawkshead Lane, Hatfield, Hertfordshire AL9 7TA UK

**Keywords:** Digital dermatitis, Heifers, Genome-wide association study, Functional enrichment analysis

## Abstract

**Background:**

Digital dermatitis (DD) is a global infectious hoof disease causing lameness in cattle. The condition has been studied extensively in milking cows. The present study deployed data from repeated clinical examinations of young heifers to develop DD phenotypes reflecting the disease prevalence and progression. The newly defined traits were then subjected to single-step genome-wide association analyses to assess their genetic profile. Data were collected during four monthly clinical examinations of 921 Holstein heifers aged less than 15 months. The M-score system was used to assess hoof health, and four phenotypes were developed on each animal: two binary traits considering the presence or absence of DD within each examination (BINR) and across all examinations (BINA) and two disease progression traits (TP1 and TP2) throughout the examination period. The latter two phenotypes were derived on heifers that were healthy at first examination (*n* = 625). Pedigree and genome-wide genotypes were available on these animals. For each trait, the ten 1-Mb windows that explained the largest proportion of the total genetic variance were used to search for positional candidate genes. Functional enrichment analysis was carried out to determine the functional candidates.

**Results:**

All traits were significantly (*P* < 0.05) heritable with heritability estimates ranging from 0.22 for TP2 to 0.32 for BINA. Our findings revealed common genomic regions between traits distributed across eight chromosomes (7, 8, 11, 12, 13, 20, 23 and 24). Functional enrichment analysis revealed multiple candidate genes including *DOCK2* and *SRF* that are known for involvement in immune response.

**Conclusions:**

Results provide novel insights into the genetic mechanism for DD development and progression in young dairy heifers.

**Supplementary Information:**

The online version contains supplementary material available at 10.1186/s12864-026-13016-y.

## Background

Digital Dermatitis (DD) is contagious and prevalent in cattle around the world [1–4]. The disease causes ulceration and/or papillomatous growths, usually located on the plantar surface of the hind foot and normally circumscribing the heel bulbs [5, 6]. The resulting lesions can be painful, leading to lameness and compromised animal health, reduced milk production, and lowered cow fertility [7–9]. The aetiology remains to be fully elucidated as DD is a polybacterial disease involving more than 20 different *Treponema* phylotypes plus other bacteria such as *Mycoplasma* [6].

Important risk factors for within-herd disease development and spread are housing conditions and management factors such as foot hygiene, herd size, and grazing and trimming routines [10]. Given that DD is endemic in many countries with a typical herd prevalence of 20–30%, whole herd treatment is often required to allow effective control and prevent reinfection [10].

Strategies to halt transmission of DD include systemic antibiotics, individual topical treatment, and the use of footbaths [3]. Strategies may differ across counties. For example, in the UK the most common approach to treating DD is the topical antibiotic application [11, 12] since the systematic use of antibiotics would require farmers to stop milking cows during treatment periods [13]. In Sweden, the recommended treatment is to directly apply bandages containing salicylic acid to the infection site [14]. Although antibiotics such as oxytetracycline and lincomycin are successfully used as individual treatments and their topical administration is considered effective, their use should be limited to avoid antimicrobial resistance and withdrawal periods for milk [15, 16]. The collective administration of antibiotics is no longer advised, and such practices are already banned by European Union policies [16]. A vaccine targeting multiple treponeme phylotypes could help DD prevention, but it has not yet been made commercially available [10].

The economic impact of DD is considerable ($64.00 to $132.96 per disease episode) due to reduced milk production, discarded milk, treatment costs, and additional labour [17, 18].

On-farm monitoring of DD records is necessary to control and minimise the spread of the disease. Recording of DD is usually based on the M-stage system [19]. Traditionally, DD recording takes place during hoof inspection at trimming in chutes where the diagnosis is dependent on the trimmers’ abilities and alertness and on whether the cows’ feet have been washed [20]. Additional diagnostic methods of DD include mirror scoring and use of a borescope on cows standing in the parlour or pen offer additional diagnostic and recording options [4, 20]. The results of any DD scoring method are based on the accuracy of the definitions of the scoring system, the tool used for the inspection, the examination conditions, and the experience of the examiners [21].

Improving the animals’ inherent capacity to resist DD development has been suggested in previous studies [9, 22, 23] that demonstrated presence of genetic variation and a trait heritability up to 0.40. Recent genomic studies have revealed promising candidate genes for DD resistance of cows at different stages of lactation and highlighted the utility of genomic breeding values in selective breeding [24–26].

Genetic studies to-date have showcased the feasibility of genetic selection to improve disease resistance towards reducing the prevalence of DD [27]. Breeding for increased DD resistance would be associated with an increase in both animal longevity and performance [23].

The focus of DD genetics research has so far been mainly placed on temporal disease profiles of milking cows. Relatively little is known about DD in maiden heifers. Gomez et al. [7] showed that heifers with repeated DD events before their first calving had a lower conception rate at first service and an increase in the number of days open after the first calving compared to healthy animals. Another study [28] investigated the impact of DD on the feedlot behaviour of beef heifers and found that rumination time was significantly greater in healthy heifers without DD lesions. Studies in the USA and Chile have reported a positive association between the introduction of new replacement heifers to a herd and DD prevalence [29–32]. Nevertheless, a Dutch study [33] found that buying replacement heifers was not a predisposing factor for DD.

The present study aims to expand knowledge on DD in young growing heifers. Our objectives were to (i) develop and examine new clinical DD phenotypes on Holstein heifers reflecting disease prevalence and progression, and (ii) assess the genetic profile of these phenotypes.

## Methods

### Data and phenotypic trait definitions

The study took place between March 2021 and June 2024 and included 921 heifers reared in a UK commercial farm. Animals were clinically examined in cohorts by two qualified veterinarians, members of our team. The feet of each individual animal were examined four times, with the interval between successive examinations ranging from 24 to 32 days. The average age of the heifers at the first examination was 296 days (min:199, max:428).

On examination date, each hind foot of the heifer was examined separately for DD lesions and scored according to the six M-stage system [19, 34], where: M0 = skin without any macroscopically visible lesions; M1 = small focal circumscribed damage of the epithelium at the skin horn border (< 2.0 cm in diameter); M2 = circumscribed ulcerative skin defect with a red or greyish surface that can have a white epithelial margin and overlong hair (> 2.0 cm in diameter); M3 = the healing stage of DD, after an M2 lesion has been covered with a scab; M4 = chronic stage of DD that is characterised by thickened or proliferative growth of the epithelium; and M4.1 = as described under M4, but with a new M1 lesion within its perimeter.

Individual animal records were used to develop four DD phenotypes. Firstly, a prevalence phenotype within each examination (BINR) was defined as follows; each heifer had four such phenotypic records, one for each examination:

- 0 = all feet were M0 in the examination
- 1 = at least one foot had a score other than M0 in the examination

Secondly, another prevalence phenotype was defined across all examinations (BINA); each heifer had one BINA record:

- 0 = all feet were M0 in all examinations
- 1 = at least one foot had a score other than M0 in at least one examination

Thirdly, a DD progression phenotype was defined on each heifer that was healthy (all feet scored as M0) at the first examination (TP1); there were 625 heifers, each with one TP1 record:

- 0 = The heifer remained healthy (M0) in all examinations
- 1 = The heifer had lesions (score other than M0) in the second and/or third examination but recovered and was healthy (M0) again at the last examination
- 2 = The heifer still had lesions at the last examination

Fourthly, a more detailed DD progression phenotype was defined on each heifer that was healthy (all feet scored as M0) at the first examination (TP2); there were 625 heifers, each with one TP1 record:

- 0 = The heifer remained healthy (M0) in all examinations
- 1 = The heifer had active lesions (score M1, M2 or M4.1) in the second and/or third examination but recovered and was healthy (M0) again at the last examination
- 2 = The heifer had active lesions (score M1, M2 or M4.1) in the second and/or third examination that turned non-active (M3 or M4) in the last examination
- 3 = The heifer still had active lesions (score M1, M2 or M4.1) at the last examination
- 4 = The heifer had non-active lesions (score M3 or M4) in the second and/or third examination but recovered and was healthy (M0) again at the last examination
- 5 = The heifer still had non-active lesions (score M3 or M4) at the last examination
- 6 = The heifer had non-active lesions (score M3 or M4) in the second and/or third examination that turned active (score M1, M2 or M4.1) in the last examination.

Table 1 summarises data for the four phenotypic trait definitions.

**Table 1:** Description of data by phenotypic trait definition

Trait abbreviation	Phenotypic trait definition	Number of animals with phenotypes	Percentage of animals by phenotypic value	Number of animals with genotypes after quality control
BINR	DD prevalence phenotype within each of four examinations; each heifer had four BINR records: 0 = all feet were M0 in the examination 1 = at least one foot had a score other than M0 in the examination	921	0: 73%, 72%, 71%, 67% 1: 27%, 28%, 29%, 33% By examination	865
BINA	DD prevalence phenotype across all examinations; each heifer had one BINA record: 0 = all feet were M0 in all examinations 1 = at least one foot had a score other than M0 in at least one examination	921	0: 57% 1: 43%	865
TP1	First DD progression phenotype on heifers that were healthy (all feet scored as M0) at the first examination; each heifer had one TP1 record: 0 = The heifer remained healthy (M0) in all examinations 1 = The heifer had lesions (score other than M0) in the second and/or third examination but recovered and was healthy (M0) at the last examination 2 = The heifer still had lesions at the last examination	625	0: 79% 1: 7% 2: 14%	624
TP2	Second DD progression phenotype on heifers that were healthy (all feet scored as M0) at the first examination; each heifer had one TP2 record: 0 = The heifer remained healthy (M0) in all examinations 1 = The heifer had active lesions (score M1, M2 or M4.1) in the second and/or third examination but recovered and was healthy (M0) at the last examination 2 = The heifer had active lesions (score M1, M2 or M4.1) in the second and/or third examination that turned non-active (M3 or M4) in the last examination 3 = The heifer still had active lesions (score M1, M2 or M4.1) at the last examination 4 = The heifer had non-active lesions (score M3 or M4) in the second and/or third examination but recovered and was healthy (M0) at the last examination 5 = The heifer still had non-active lesions (score M3 or M4) at the last examination 6 = The heifer had non-active lesions (score M3 or M4) in the second and/or third examination that turned active (score M1, M2 or M4.1) in the last examination.	625	0: 79% 1: 4% 2: 3% 3: 6% 4: 3% 5: 4% 6: 1%	624

### Genetic data and quality control

All heifers had been genotyped with a 65 K Axiom_VM2V2 Single Nucleotide Polymorphism (SNP) genotyping array (Genetic Visions-ST™). Only autosomal SNP markers were considered in the present study. Quality control was performed firstly at animal sample and secondly at SNP marker level. We excluded animals with call rate lower than 0.90. We also excluded SNPs with call rate lower than 0.90, minor allele frequency lower than 0.05 and linkage disequilibrium (LD) manifested in r^2^ >0.90 (window size: 50 SNPs, increment: 5 SNPs). Quality control was performed in PLINK [35]. After these filters, a total of 39,816 SNPs were retained for 865 and 624 animal samples pertaining to the DD prevalence and progression phenotypes, respectively (Table 1).

A comprehensive, quality-assured pedigree dataset was also available for the study population, generated from the UK official national genetic evaluation (Edinburgh Genetic Evaluation Services, EGENES). A pedigree comprising 1,650 animals and spanning three generations of ancestors of the phenotyped animals was extracted and used in the ensuing data analyses.

### Principal component and linkage disequilibrium analyses

The genomic structure of the studied population was explored with a principal component (PC) analysis of the animals’ genotypes. The top 20 PCs were estimated with the ‘—pca’ command in PLINK [35]. The first two PCs were plotted on the two-dimensional space to assess genetic patterns in the studied population (Supplementary Fig. 1, Additional File 1). Plot was constructed using the ggplot2 R package [36]. The first PC was then retained to be included in the genomic association analyses in order to account for population structure.

We estimated LD between all SNPs using PLINK [35]. Subsequently, LD decay was derived and plotted using the packages dplyr [37], stringr [38], ggplot2 [36] in R (Supplementary Fig. 2, Additional File 2). Based on the average distance where LD between markers was halved, we determined the size of 1 Mb for the ensuing window-based genomic analyses.

### Single-step genome-wide association (ssGWA) analyses

The animal repeatability model (1) was used to analyse the repeated records of the BINR phenotype at the four examinations per animal:

1 $$y=\mathrm{X}\beta+\mathrm{Zg}+\mathrm{Wpe}+e$$

where y is the vector of phenotypic values, β is a vector of fixed effects including the scorer, calendar season of heifer’s birth, cohort of animal clinical examination, age of heifer at examination, and the first PC from the PC analysis of genotypes; g is the vector of the random animal additive genetic effects following var(g) ~ N(0, Hσ^2^_g_) distribution, where H is the matrix of additive genetic relationships incorporating both pedigree and genomic data [39] and σ^2^_g_ is the additive genetic variance; pe is a vector of the random permanent environmental effect with var(pe) ~ N(0, Iσ^2^_pe_), where σ^2^_pe_ is the permanent environmental variance and I is the identity matrix; e is a vector of random residuals with distribution of var(e) ~ N(0, I σ^2^_e_) in which I is the identity matrix and σ^2^_e_ is the residual variance; X, Z and W are incidence matrices relating records to β, g and pe, respectively.

The H matrix in model (1) was generated as follows [39–41]:

$$H^{-1}\:=\begin{bmatrix}0&\:0\\\:{0\:G}^{-1}&\:{-A}_{22}^{-1}\end{bmatrix}+A^{-1}$$

where $\:{A}^{-1}$ is the inverse of the numerator pedigree relationship matrix encompassing all animals in the pedigree file, $\:{A}_{22}^{-1}$ is the inverse of the submatrix of *A* pertaining to the genotyped animals and $\:{G}^{-1}$ is the inverse of the genomic relationship matrix of the genotyped animals. The *G* matrix was constructed as proposed by VanRaden [42].

The mixed model applied for the analyses of the other three DD phenotypes (BINA, TP1 and TP2) had the same effects as model (1) except for removing the random permanent environmental effect because each animal had a single record of these traits. Age of heifer at examination was also removed from the analyses of TP1 and TP2 since the effect on these traits was not statistically significant (*P* > 0.05).

All analyses were conducted with the BLUPF90 family software [43, 44].

Heritability of each trait was calculated as the ratio of the estimated additive genetic variance to the phenotypic variance. Repeatability of BINR was derived as the ratio of the sum of the estimated additive genetic and permanent environmental variance to the phenotypic variance.

Genomic breeding values were estimated for each animal and phenotypic trait. Individual SNP effects and *P*-values were then obtained by back-solving genomic breeding values using POSTGSF90 [44, 45]. Corresponding *P*-values were subsequently corrected for multiple comparisons using the Bonferroni correction method. Significance at the genome-wide level required a *P*-value lower than the threshold of 1.26E-06 (0.05/39,816), while a suggestive SNP had a *P*-value lower than 2.51E-05 (1/39,816).

We also constructed quantile-quantile (Q-Q) plots to characterise the extent to which the observed distribution of the test statistic followed the expected (null) distribution. These plots in combination with the estimation of the genomic inflation factor (λ) were used to confirm absence of bias arising from population structure or the analytical approach [46]. The Manhattan and Q-Q plots were constructed using the CMplot package (https://github.com/YinLiLin/R-CMplot) in R (http://www.r-project.org/).

In addition to the single-locus analyses, a 1 Mb sliding-window approach was implemented using POSTGSF90 to calculate the corresponding proportion of genetic variance explained by each genomic window. The corresponding Manhattan plots were constructed with the POSTGSF90 software.

### Detection of positional candidate genes and functional enrichment analysis

The genomic windows that included statistically significant individual SNPs from the single-locus ssGWA described above and the ten genomic windows that accounted for the highest proportion of the trait genetic variance were considered for presence of positional candidate genes [24, 47]. These genes were retrieved from the Ensembl BioMarts hub [48] using the ARS-UCD1.2 bovine genome assembly.

Gene Ontology (GO) Biological Process (BP) enrichment analysis was then applied to the positional candidate genes using the g: GOSt tool of the g: Profiler web server (https://biit.cs.ut.ee/gprofiler/gost [49]). We searched for GO BP significantly enriched annotations of *Bos taurus* input genes with FDR *P*-values lower than 0.05 [50]. Each resulting significantly enriched GO BP was further examined on the web with AmiGO2 [7]. GO BPs associated with the immune system were considered as functionally relevant to the phenotypic traits under study.

## Results

### Lesion prevalence and progression

The hind feet of 921 young Holstein heifers were clinically examined for DD lesions four times in approximately 30-day intervals. DD prevalence within examination (BINR) was 27–33% (Table 1), whilst across all four examinations (BINA) DD prevalence was 43%. Amongst the 625 heifers that entered the study healthy, 21% developed lesions (TP1) of which one third recovered by the last examination (Table 1). For TP2, the highest profile proportion (6%) was observed for heifers that developed active lesions and had not recovered by the last examination (Table 1).

### Genetic parameters

Estimates of variance components and genetic parameters are summarised in Table 2. All DD phenotypes exhibited significant genetic variation with heritability estimates significantly greater than zero (*P* < 0.05), ranging from 0.22 (SE = 0.09) for TP2 to 0.32 (SE = 0.07) for BINA. Additionally, BINR was highly repeatable across the four examinations with a repeatability estimate of 0.59 (SE = 0.02).

**Table 2:** Estimates of additive genetic variance (Vg), permanent environmental variance (Vpe), residual variance (Ve), repeatability (r) and heritability (h^2^) (± standard errors) for the digital dermatitis prevalence (BINR and BINA) and progression (TP1 and TP2) traits

Trait^1^	Vg	Vpe	Ve	*r*	h^2^
BINR	0.05 ± 0.01	0.07 ± 0.009	0.08 ± 0.002	0.59 ± 0.02	0.26 ± 0.05
BINA	0.08 ± 0.02	NA	0.16 ± 0.01	NA	0.32 ± 0.07
TP1	0.11 ± 0.05	NA	0.37 ± 0.04	NA	0.23 ± 0.09
TP2	0.67 ± 0.28	NA	2.33 ± 0.25	NA	0.22 ± 0.09

^**1**^Complete trait definition in Table 1

### Single-locus genomic analyses

The Q-Q plots and the genomic inflation factor (λ) estimates from the genomic analyses are given in Supplementary Fig. 3 (Additional File 3). The λ estimates (0.97–1.01) show no evidence of systematic bias attributed to population structure or analytical approach [46] suggesting that inclusion of the H matrix and PC in the model of analysis successfully mitigated possible substructure issues.

**Fig. 1:**
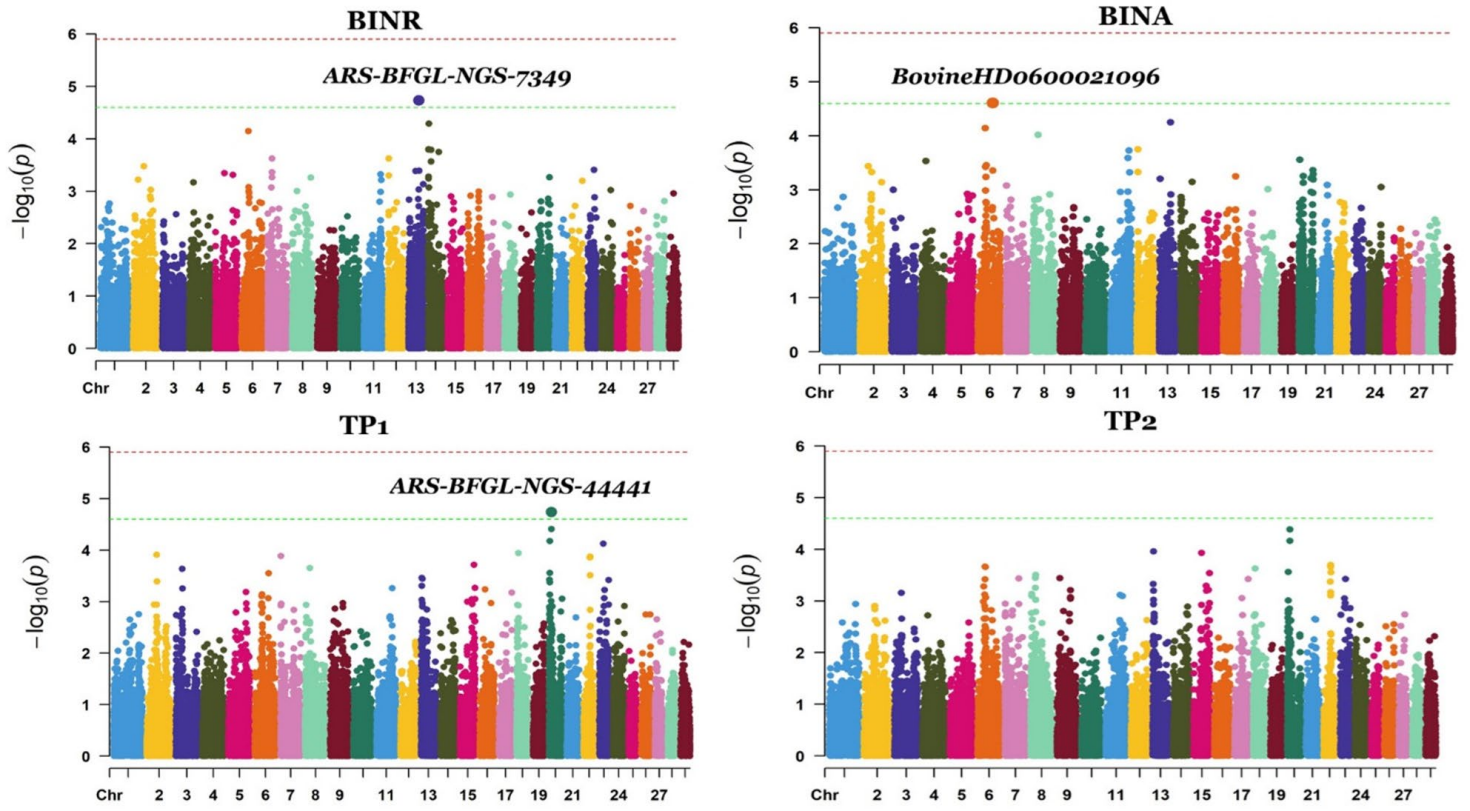
Manhattan plots illustrating the −log_10_(*P*-values) of Single Nucleotide Polymorphisms across the 29 autosomes for the digital dermatitis traits of study (BINR, BINA, TP1 and TP2). The red horizontal line represents the genome-wide significance threshold, while the green horizontal line denotes the suggestive significance threshold

Figure 1 presents the profiles of the SNP *P*-values (expressed as −log_10_ values) for the four studied traits. No single marker reached genome-wide significance after the Bonferroni correction. Nevertheless, three suggestive SNPs were detected for BINR, BINA and TP1. Specifically, *ARS-BFGL-NGS-7349* on chromosome 13 (56,356,164 bp, *P*-value = 1.863397e-05) was associated with BINR, *BovineHD0600021096* on chromosome 6 (74,177,762 bp, *P*-value = 2.463959e-05) with BINA and *ARS-BFGL-NGS-44,441* on chromosome 20 (12,386,636 bp, *P*-value = 1.833697e-05) with TP1.

### Genomic window analyses and positional candidate genes

**Fig. 2:**
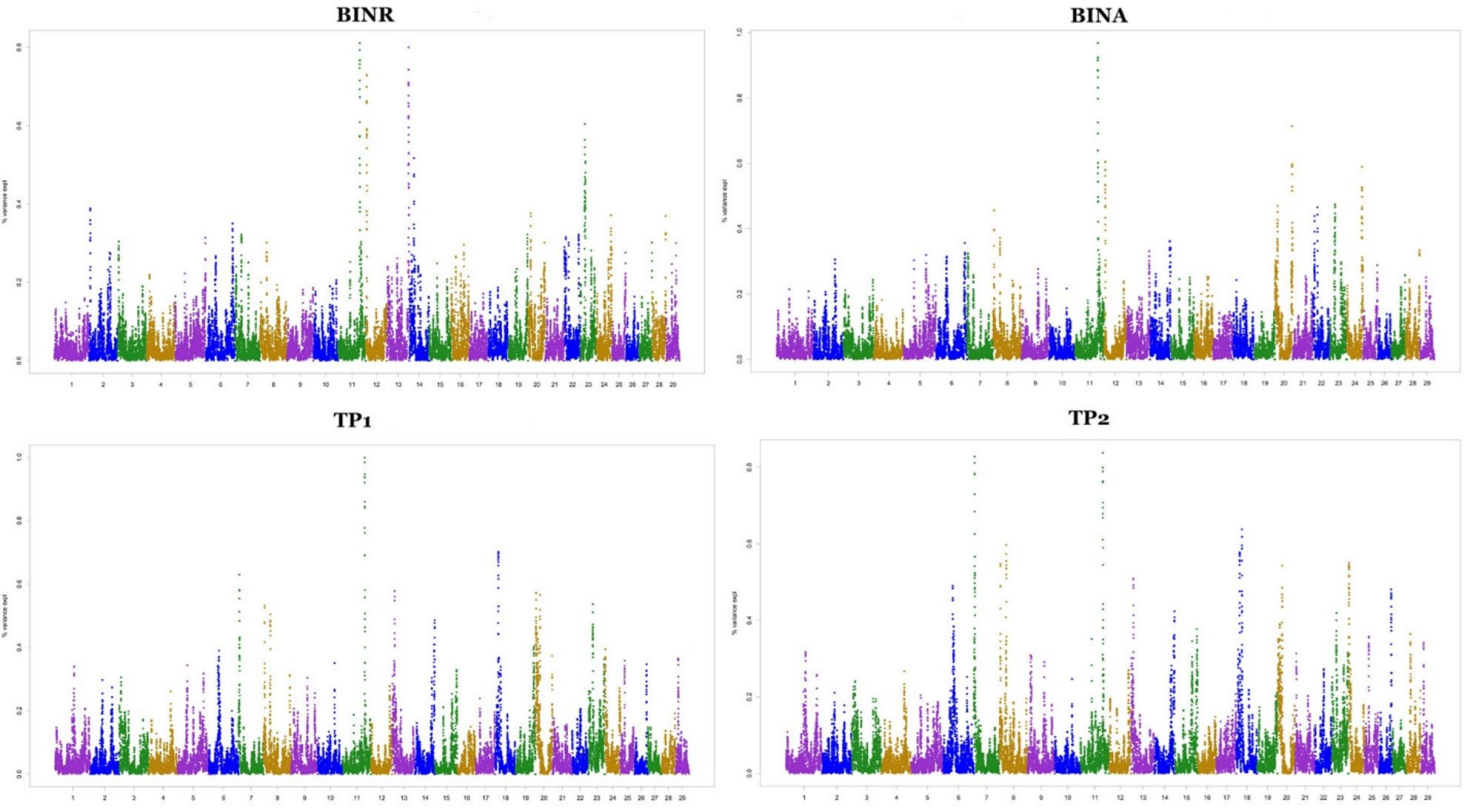
Manhattan plots of the genetic variance explained by 1 Mb sliding window for the digital dermatitis traits of study (BINR, BINA, TP1 and TP2)

Figure 2 includes the Manhattan plots with respective percentages of genetic variance explained by 1-Mb windows per trait. The 1-Mb window size was determined in the linkage disequilibrium (LD) decay analysis of the animal genotypes (Supplementary Fig. 2 (Additional File 2)).

The top ten genomic windows with the highest percentage of genetic variance explained and the corresponding positional candidate genes are summarised in Supplementary Table 1 (Additional File 4). Notably, one of the top ten genomic windows included the *ARS-BFGL-NGS-44,441* marker that was suggestively associated with TP1 in the single-locus analysis, but none of them included *ARS-BFGL-NGS-7349* or *BovineHD0600021096* that were linked to BINR and BINA, respectively. We further searched if *ARS-BFGL-NGS-7349* and *BovineHD0600021096* were located within a gene, but none was found. Nevertheless, a total of 177 positional candidate genes (Supplementary Table 1, Additional File 4) were identified within the 1-Mb genomic windows.

For all traits, each of the top ten windows explained less than 1% of the genetic variance (Supplementary Table 1, Additional File 4). The highest was a 1-Mb window on chromosome 11 (85,075,162 − 86,061,595 bp) that accounted for 0.99% of the TP2 variance. One gene (*TRIB2: tribbles pseudokinase 2*) was detected within this region (Supplementary Table 1, Additional File 4). The same window also explained 0.84% of the TP1 genetic variance. An additional region located on chromosome 11 (85,231,543 − 86,216,179 bp) explained the highest percentage of the total genetic variance (0.97%) for BINA and 0.81% of the BINR variance. This region harboured two genes (*TRIB2: tribbles pseudokinase 2*,* LPIN1: lipin 1*). Beyond chromosome 11, common genomic windows between TP1 and TP2 were also detected on chromosomes 7 (3,108,200 -4,068,721 bp), 8 (24,718,037 − 25,709,945 bp, 3,389,399 -4,388,296 bp), 13 (8,767,133 -9,734,692) and 20 (12,376,841 − 13,357,629 bp). For BINA and BINR, there were five common regions on chromosomes 11 (85,231,543 − 86,216,179 bp), 12 (3763326–4735467 bp), chromosome 20 (7049088–8032579 bp), chromosome 23 (15241709–16218110 bp) and chromosome 24 (58981347–59971040 bp). One common region was detected on chromosome 23 (15,241,709 − 16,218,110 bp) for BINR, BINA and TP2.

### Functional enrichment analysis

All the afore-mentioned positional candidate genes (*n* = 177) were recognised by the g: Profiler tool. Functional enrichment analysis identified 131 significantly enriched Gene Ontology (GO) Biological Processes (BPs) with 172 participating genes (Supplementary Table 2, Additional File 5). According to AmiGO2 (Supplementary Fig. 4, Additional File 6), the positive thymic T cell selection BP (GO:0045059) is an immune system process with *DOCK2 (dedicator of cytokinesis 2)* and *SRF (serum response factor)* genes.

## Discussion

The present study addressed DD prevalence and progression in growing maiden heifers aged less than 15 months. Novel animal phenotypes were developed, and their genetic profile was investigated. Understanding the prevalence of foot lesions within a herd is the starting point in identifying the factors contributing to lameness and developing prevention strategies [51]. Beyond management and environmental influences [52], host genetics also affect the DD risk, and this was the focus of the present study.

We explored disease prevalence during four consecutive rounds of detailed clinical foot examination at approximately 4-week intervals in 921 Holstein heifers. We also examined the foot health profile across the four examinations of animals that were healthy at the beginning (*n* = 625) of the study and defined novel disease progression phenotypes for these heifers.

The percentage of afflicted individuals in each examination ranged from 27% to 33%, whilst across all four examinations, DD period prevalence was 43%. This is broadly consistent with a previous study on older pregnant Holstein heifers (average age of 21 months) reared in a US herd that reported DD incidence of 32% [34]. Results also fall within the range of estimates reported on dairy cows [24, 53] suggesting that the magnitude of the DD problem is at least as large among the young stock as in the milking herd. The challenges of implementing effective interventions for lameness control in UK dairy heifers have been analysed and documented [54], thereby adding to the conviction that additional measures are warranted to improve foot health of young cattle.

Amongst the heifers that were healthy at the beginning of the present study, 21% developed DD lesions in the ensuing four months. Of them, one third fully recovered, whilst two thirds still had lesions at the end of the study. The latter were equally divided between heifers with active (M1, M2 or M4.1) and chronic non-active (M3 or M4) lesions. These observations demonstrate the likelihood of differences among individual heifers in disease dynamics. Differences in disease progression among individuals was also reported in the previous US heifer study [27].

We then deployed the ssGWA approach, which simultaneously combines all phenotypic, genotypic and pedigree data available [44] to examine the genetic component of the heifer DD traits. We derived variance component and genetic parameter estimates and investigated single-locus SNP and window-based genomic associations with the phenotypic traits of study. All DD traits exhibited significant (*P* < 0.05) genetic variation suggesting that genetic improvement based on selective breeding is feasible and may decisively contribute to controlling disease prevalence and progression in dairy heifers.

Our heritability estimates were 0.26–0.33 and 0.22–0.23 for the DD prevalence and progression traits, respectively. These results are comparable to pedigree heritability estimates reported by Schöpke et al. [34] in the only previous genetic study on dairy heifer DD that we are aware of. Results are also within the broad range of DD heritability estimates in milking cows [22, 24] suggesting a similar extent of additive genetic influence in young and older dairy cattle.

Our genomic association results revealed a largely polygenic nature in all the studied DD phenotypes. The largest proportion of trait genetic variance explained by a 1 Mb window was just under 1% and the top ten genomic windows by amount of variance explained were dispersed across 14 autosomes.

Nevertheless, certain interesting regions and candidate genes were identified. Specifically, one region on chromosome 6 (37,804,003–38,789,984 bp) associated here with DD progression overlapped with a previously reported candidate region for DD incidence in dairy cows during their dry period and around the peak milk production stage of lactation [24].

Another region on chromosome 13 (8,767,133 -9,734,692 bp) that was also associated with DD progression in our study overlapped with a reported genomic window affecting foot health of dairy cows in early stage of lactation [24]. This region harbours the *MACROD2 (mono-ADP ribosylhydrolase 2)* candidate gene that has been also associated with resistance to bovine tuberculosis [55] and anogenital distance in cattle [56].

Moreover, a region on chromosome 24 (58,981,347 − 59,971,040 bp) that was associated with DD prevalence in our study overlapped with a region linked to DD in dairy cows during the dry period [24]. Two candidate genes, *MC4R* (*melanocortin 4 receptor*) and *CDH20* (*cadherin 20*), are located in this region that have also been reported for association with growth traits in Madrasin [57] and Simbrah cattle [58], respectively.

The identification of the above-mentioned overlapping regions on chromosomes 6, 13 and 24 suggest a partly similar genetic control of DD resistance in young heifers and older milking cows. Future studies to fully address this would be useful. A strong positive genetic correlation of the traits in heifers and cows would facilitate genetic improvement programmes towards enhancing the early genetic capacity for disease resistance with long-lasting effects.

Furthermore, a region on chromosome 14 (80,988,420 − 81,959,530 bp) for DD progression and another on chromosome 23 (16,313,445 − 17,302,126 bp) for prevalence could be of particular interest as they harbour the *DEPTOR* (*DEP domain containing MTOR interacting protein*) and *BICRAL (BICRA like chromatin remodelling complex associated protein)* genes, respectively, that were previously associated with the digital cushion thickness, a structure that protects the hoof from injuries and lesion development in cattle [59].

Application of functional enrichment analysis revealed *DOCK2 and SRF* as functional candidate genes for resistance to DD. In cattle, *DOCK2* gene has been reported for specific DD diagnosis [60]. In humans, *DOCK2* is mainly expressed in haematopoietic tissues, especially immune cells, and plays an important role in the regulation of the immune system and the serum response factor, thereby affecting the expression of smooth muscle cell markers [61]. In mice, postnatal loss of the serum response factor in keratinocytes reportedly results in the development of psoriasis-like skin lesions [62]. These lesions were characterised by inflammation, hyperproliferation, and abnormal differentiation of keratinocytes as well as disruption of the actin cytoskeleton [62]. In the skin lesions of *SRF* mutant mice, a previous study [62] found suprabasal expression of the β1 integrin subunit in the interfollicular epidermis that is associated with hyperproliferation and perturbed differentiation of keratinocytes and skin inflammation, as in psoriasis [63].

Additional plausible candidate revealed in our functional enrichment analysis included the *TRIB2* and *LPIN1* genes that have been associated with inflammation and disease in humans. Specifically, diverse roles of *TRIB2* have been reported in neurological disorders, metabolic diseases, autoimmune and inflammatory diseases, arthritis, and multiple cancers [64], while *LPIN1* has been related with inflammation and immune response in sepsis incidents [65].

In closing, the multifactorial nature of DD in heifers warrants multifaceted approaches to addressing the issue. Successful DD prevention and control programmes already in place for milking cows should expand to include rearing-age heifers aiming to improve animal health and welfare and reduce recurrence rates during the first lactation. Our results may collectively offer input to the selective breeding component of such programmes. A previous study [27] highlighted that digital dermatitis genetic indexes aid herd management of DD in cows, and suggest that breeding for resistance to DD, alongside environmental and management control practices, could reduce the prevalence of the disease.

Active animal surveillance on the farm is recommended to generate sufficient records for genetic evaluation, while informing management practices including the timely administration of treatment to infected individuals. A consolidated practice of animal surveillance and inspection could also benefit from housing the young stock together with the lactating cows, a practice that has been shown to reduce incidence of DD in dairy cattle [66].

## Conclusions

We sought to define new traits for DD in young heifers capturing disease dynamics at specific timepoints and in transition across distinct stages of clinical examination. Genetic analyses revealed the presence of genetic variation rendering the studied traits amenable to improvement by selective breeding. Genetic improvement may manifest in enhancing the inherent resistance of animals to develop DD lesions as well as their capacity to recover if they become afflicted. Functional candidate genes were identified that may contribute to understanding the genetic mechanisms preventing DD lesions and/or promoting the healing process in growing heifers. Results may also inform future studies on the discovery of the causal genetic variants controlling DD development and progression in the early life of dairy cattle.

## Supplementary Information

Additional file 1: Supplementary Figure 1. Two-dimensional plot of the first two principal component (PC) axes of genetic variation (labelled PC1 and PC2) of heifer genotypes. The percentage of variance each PC axis explained is also provided. Each point represents an individual genotype, and points are coloured by scorer.

Additional file 2: Supplementary Figure 2. Linkage disequilibrium decay between markers in the examined heifers.

Additional file 3: Supplementary Figure 3. Q-Q plots and the genomic inflation factor (λ) estimates from the genomic analysis of BINR, BINA, TP1 and TP2.

Additional file 4: Supplementary Table 1. Top ten genomic windows with the highest percentage of genetic variance explained per trait. Positional candidate genes are also given for each genomic window.

Additional file 5: Supplementary Table 2. Significantly enriched Gene Ontology (GO) Biological Process (BP) terms and the respective genes.

Additional file 6: Supplementary Figure 4. GO hierarchical structure for the positive thymic T cell selection BP. This GO tree was created and extracted with AmiGO2.

## Data Availability

The data that support the findings of this study are available from Scotland’s Rural College (SRUC), but restrictions apply to certain data (including the animal genotypes) that were used under license for the current study. Data can become available from the corresponding author upon reasonable request and with permission of SRUC. We provide a description of data in Table 1 of the manuscript. The positions of SNPs and genes were based on the ARS-UCD1.2 bovine genome assembly. The profiles of the SNP *P*-values (expressed as −log_10_ values) for the studied traits are provided in Fig. 1. The Q-Q plots and the genomic inflation factor (λ) estimates from the genomic analyses are given in Supplementary Figure 3 (Additional File 3). The list of positional candidate genes and genomic window per trait are provided in Supplementary Table 1 (Additional File 4). Finally, we provide the Manhattan plots with respective percentages of genetic variance explained by 1-Mb windows per trait in Fig. 2.

## Abbreviations

DD: digital dermatitis
BINR: prevalence phenotype within each examination
BINA: prevalence phenotype across all examinations
TP1: First DD progression phenotype
TP2: Second DD progression phenotype
